# Cell type specification and diversity in subpallial organoids

**DOI:** 10.3389/fgene.2024.1440583

**Published:** 2024-09-26

**Authors:** Narciso Pavon, Yubing Sun, ChangHui Pak

**Affiliations:** ^1^ Department of Biochemistry and Molecular Biology, University of Massachusetts Amherst, Amherst, MA, United States; ^2^ Graduate Program in Neuroscience and Behavior, University of Massachusetts Amherst, Amherst, MA, United States; ^3^ Department of Mechanical and Industrial Engineering, University of Massachusetts Amherst, Amherst, MA, United States

**Keywords:** brain region-specific organoids, neural organoids, shh, fgf, patterning, subpallium, iPSCs, MSNs

## Abstract

Neural organoids have emerged as valuable tools for studying the developing brain, sparking enthusiasm and driving their adoption in disease modeling, drug screening, and investigating fetal neural development. The increasing popularity of neural organoids as models has led to a wide range of methodologies aimed at continuous improvement and refinement. Consequently, research groups often improve and reconfigure protocols to create region-specific organoids, resulting in diverse phenotypes, including variations in morphology, gene expression, and cell populations. While these improvements are exciting, routine adoptions of such modifications and protocols in the research laboratories are often challenging due to the reiterative empirical testing necessary to validate the cell types generated. To address this challenge, we systematically compare the similarities and differences that exist across published protocols that generates subpallial-specific organoids to date. In this review, we focus specifically on exploring the production of major GABAergic neuronal subtypes, especially Medium Spiny Neurons (MSNs) and Interneurons (INs), from multiple subpallial organoid protocols. Importantly, we look to evaluate the cell type diversity and the molecular pathways manipulated to generate them, thus broadening our understanding of the existing subpallial organoids as well as assessing the *in vitro* applicability of specific patterning factors. Lastly, we discuss the current challenges and outlook on the improved patterning of region-specific neural organoids. Given the critical roles MSN and IN dysfunction play in neurological disorders, comprehending the GABAergic neurons generated by neural organoids will undoubtedly facilitate clinical translation.

## 1 Introduction

### 1.1 A brief introduction to the subpallium and stem cell derived subpallial organoids

**FIGURE 1:**
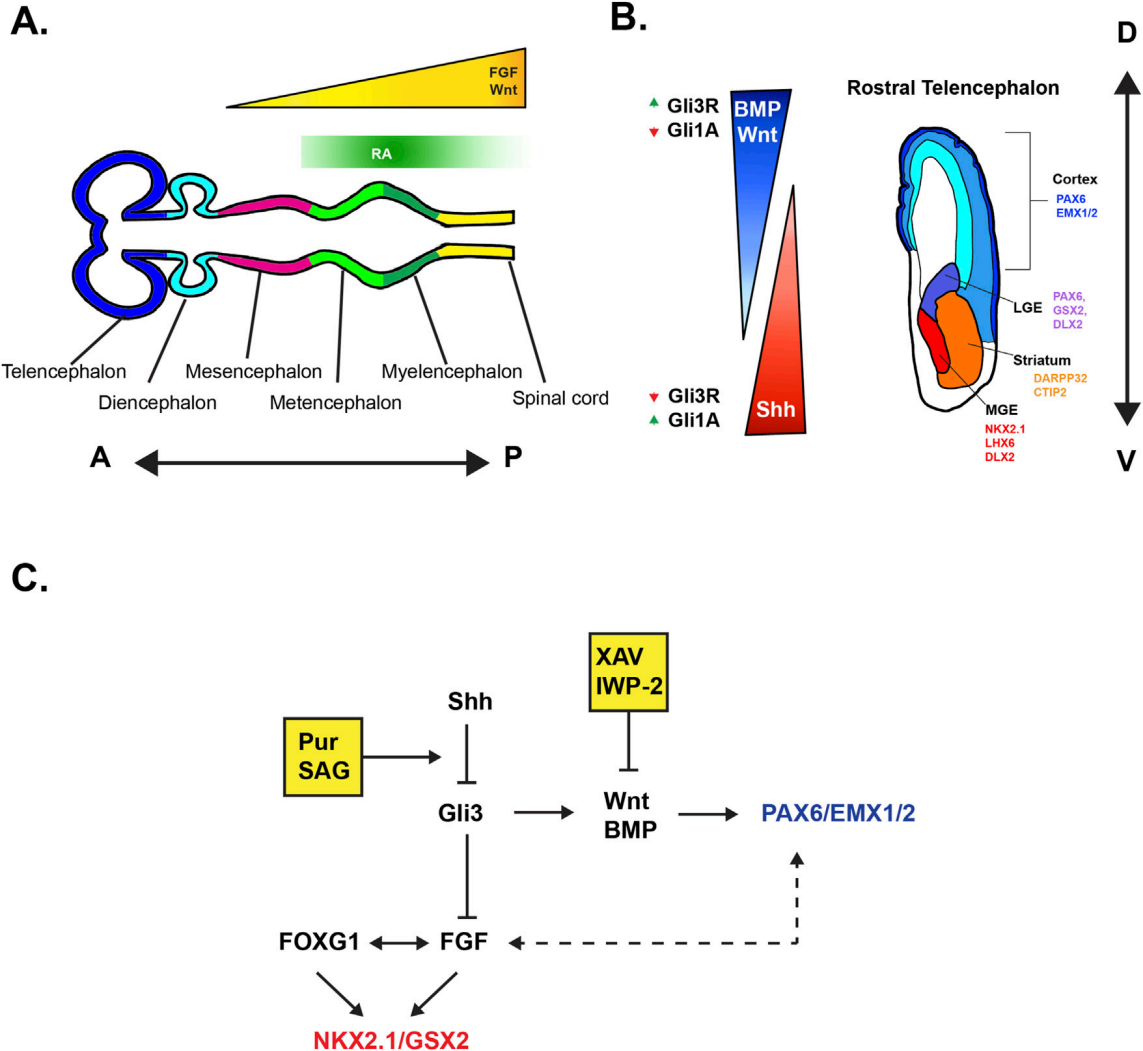
Visual schematics of morphogen effects of on developmental axes **(A)** Anterior-Posterior patterning of the secondary brain vesicles stage in embryonic development driven in large part by FGF, Wnt, and RA. Note that FGF expression from the ANR of the telencephalon is not pictured here. **(B)** Dorsal-Ventral patterning of the telencephalon. Illustrated coronal section of the fetal telencephalon depicting the role of morphogens in patterning. Up and down arrows indicate levels of Gli genes in reference to morphogenic gradients. Modified from Pavon et al. (2024). **(C)** Simplified visualization highlighting the bi-directional roles of FOXG1 and FGF in ventralization (NKX2.1/GSX) and the indirect cortical expansion (PAX6) visualized by the dotted bidirectional arrows. Yellow boxes depict the influence of common small molecule Shh agonists (Pur, SAG) and Wnt antagonists (XAV939, IWP-2).

The subpallium encompasses the rostral region of the telencephalon that is ventral to the cerebral cortex. Early in neurodevelopment, the fetal subpallium is established in large part from the coordinated gradients of key morphogens such as FGF and Shh, as evidenced by classical studies performed in chick embryos and mouse models (Figure 1A) (Ericson et al., 1995; Shimamura and Rubenstein, 1997; Houart et al., 1998). At this early time point, the morphogenic cues give rise to the transient subpallial structures referred to as the ganglionic eminences (GE’s) (Figure 1B) (Marín and Rubenstein, 2001; Rallu et al., 2002). The GE’s are crucially responsible for birthing a large majority of the cortical interneuron (IN) population, olfactory bulb (OB) interneurons, and establishing the striatum. At the most ventral end of the GEs, the Medial Ganglionic Eminence (MGE) will ultimately give rise to ∼70% of the cortical IN population, whereas with the more dorsally located, Caudal Ganglionic Eminence (CGE), contributes to ∼30% of cortical INs. The cortical fated INs of the GEs tangentially migrate towards the cortex through the broadly defined superficial migratory streams (SMS) (Bastaki et al., 2017) or the deep migratory streams (DMS) (Lavdas et al., 1999; Wichterle et al., 2001; Tanaka et al., 2003; Bastaki et al., 2017; Lim et al., 2018a). The Lateral Ganglionic Eminence (LGE), positioned rostral to the CGE, establishes the striatum and populates it with Medium Spiny Neurons (MSNs), which constitute ∼90% of cells in the striatum (Kawaguchi, 1997). Striatal INs are partially composed of CGE/MGE derived INs and make up ∼10% of cells in rodent models and up to ∼20% in primates (Marín et al., 2000; Wu et al., 2000). The LGE derived INs specifically migrate through the rostral migratory stream towards the OB (Carlos et al., 1996; Stenman et al., 2003).

**FIGURE 2:**
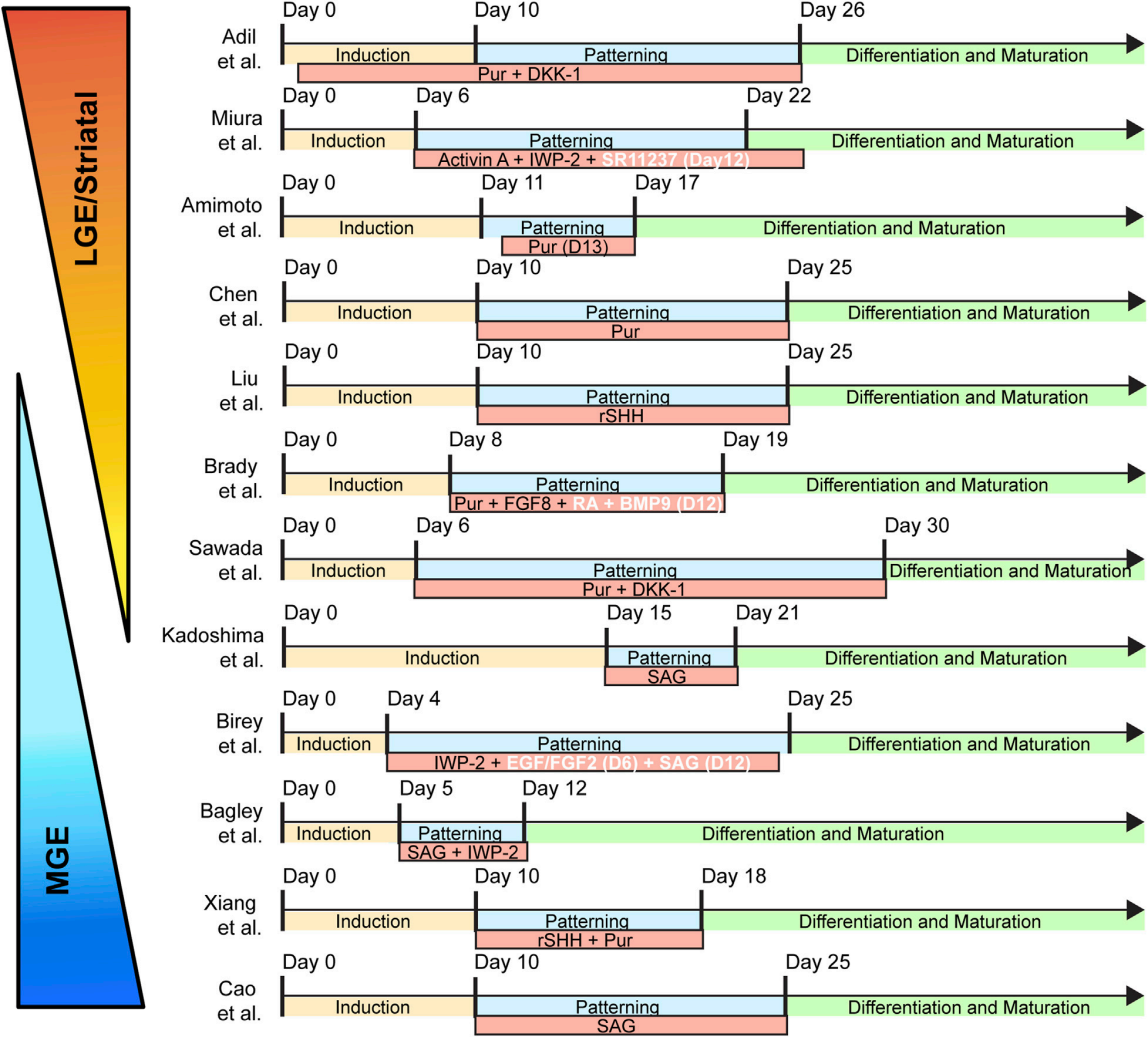
Timing and patterning schemes of published subpallial organoids. The subpallial protocols are broken down to three broadly defined categories: “induction”; “patterning”; and “differentiation and maturation.” “Induction” refers to the initial 3D aggregation and their guidance to a neuroepithelial fate using a combination of dual Smad inhibition with optional WNT inhibition. “Patterning” indicates the addition of ventralizing morphogens, usually in the form of recombinant proteins or small molecules. “Differentiation and Maturation” signals the medium conditions optimized for long-term organoid culturing. Abbreviations: Pur (Purmorphamine; Shh agonist), DKK-1 (Dickkopf1; Wnt antagonist), Activin A (Growth factor), IWP-2 (Wnt antagonist), SR11237 (RXRG agonist), rSHH (Recombinant Sonic Hedgehog), FGF8 (Fibroblast Growth Factor 8), BMP9 (Bone Morphogenetic Protein 9), SAG (Smoothened Agonist; Shh agonist), EGF (Epidermal Growth Factor), FGF2 (Fibroblast Growth Factor 2).

Recently, researchers have attempted to model the fetal subpallium by leveraging the pluripotency of human pluripotent stem cells (hPSCs) to form self-assembling 3D forebrain organoids (Table 1; Figure 2). While protocols amongst different groups can vary, most subpallial protocols use recombinant proteins and/or small molecules to stimulate the pathways that ventralizing morphogens are known to activate (i.e., Shh, FGF). The end products are a wide variety of subpallial organoids with varying degrees of ventralization and a mixture of cells representing multiple subpallial structures. In this review, we examine the neuronally specified cellular diversity of several subpallial organoids published to date with a focus on GABAergic populations.

**TABLE 1:** Summary of selected neural organoid protocols with specific patterning manipulations to LGE/Striatal and MGE subpallial subregions. Cell type identification used the reported gene expressions of each protocol to broadly classify the types of cells that might be found within each organoid.

References	Characterization	Cell lines	Patterning molecules	Cell type identification	Takeaways
Adil et al. (2018)	IHC, qPCR, Action potential analysis using voltage-sensitive dyes, Cell transplantation	H1 ESCs (WiCell), H9 ESCs (WiCell), 8FLVY6C2 iPSCs	D2-26: Pur (0.65 µM) + DKK-1 (100 ng/ml)	**Early markers:** Early LGE (GSX2, OTX2), Forebrain (FOXG1) **Late markers:** Late LGE (FOXP1, FOXP2), Pan-neuronal (MAP2), MSN (DARPP32/CTIP2/GABA/CB), Astrocyte (GFAP)	Showed a higher efficiency of MSN generation when starting in a 3D platform. At d60, 3D organoids had 69% of cells displaying action potentials
Miura et al. (2020)	IHC, RT-qPCR, scRNA-seq, Optogenetics, Calcium imaging, Whole cell patch clamp electrophysiology	Five control iPSCs derived from healthy donors, three iPSCs derived from 22q13.3DS patients	D6-22: Activin A (50 μg/ml) + IWP-2 (2.5 µM) D12-22: SR11237 (100 nM)	**Early markers:** Early LGE (GSX2, MEIS2, HOPX), Early striatal (CTIP2), Proliferating cells (TOP2A), Forebrain (FOXG1), Early ventral forebrain (DLX1/2, ASCL1) **Late markers:** GABAergic neurons (GAD1, GAD2, STMN2, SYT1), Glutamatergic neurons (SLC17A7, SLC17A6), Late ventral forebrain (DLX5/6), INs (SST, CB, CR, TH, NOS1, NPY), Mature Neurons (MAP2), MSN (DARPP32, CTIP2, NeuN), D1-MSN (DRD1), D2-MSN (DRD2), Striasome (TAC1, PENK), Astrocyte (GFAP, AQP4), Oligodendrocyte (OLIG2, SOX10, MBP)	Neurons in striatal organoid form abundant dendritic spines with ephys confirmed activity like that described in MSNs. Observed increased calcium spikes in cortico-striatal assembloid
Amimoto et al. (2021)	ICC/IHC, qPCR, MEA	1231A3 iPSCs	D13-17: Pur (0.5 µM)	**Early markers:** Early LGE (GSX2), Neural progenitors (SOX2, Ki67, pH3), Early ventral forebrain (DLX1/2, ASCL1), Forebrain (FOXG1, OTX2), Early striatal (CTIP2), **Late markers:** Mature neurons (MAP2, DCX, TUBB3, SYN1), MSN (DARPP32, CTIP2, GABA, CB)	With long-term culture rosette structures disappeared and mature markers were more abundant. Spontaneous activity recorded ∼ D64 by MEA.
Chen et al. (2022)	IHC, RT-qPCR, scRNA-seq	H9 ESCs (WiCell), 8-12 iPSC derived from healthy donors	D10-25: Pur (0.65 µM)	**Early markers:** Early LGE (GSX2, MASH1, MEIS1/2), Early ventral forebrain (DLX1/2, ASCL1), Early striatal (CTIP2) Forebrain (FOXG1, OTX2), Neuroectoderm (PAX6, SOX1), Neural progenitors (SOX2, Ki67), **Late markers:** Late LGE (FOXP2), Mature Neurons (MAP2, NeuN, STMN2, DCX, TUJ1, PSD95, BSN), Developing MSN (MASH1, CTIP2, GABA), Developing MSN 2 (DARPP32, MAP2, NeuN), MSN (DARPP32, CTIP2, GABA, NeuN, CB, CR), GABAergic neurons (GAD1, GAD2), Glutamatergic neurons (SLC17A6, SLC17A7), Striasome (MOR1), Astrocyte (AQP4, SLC1A3), Oligodendrocyte (OLIG2, MBP),	Stiatal organoids were capable of self-organized regionalization into striasome and matrix compartments. Confirmed the establishment of projections between cortico-striatal and midbrain-striatal assembloids respectively
Liu et al. (2022)	IHC, RT-qPCR, scRNA-seq, Western blot	H9 ESCs (WiCell), IMR90-4 iPSCs (WiCell), HDUE003 iPSCs	D10-25: SHH (20–200 ng/mL)	**Early markers:** Early LGE (GSX2, PAX6, MEIS2), Forebrain (FOXG1), Early ventral forebrain (DLX2, ASCL1), Neural progenitors (SOX2, HES1, Ki67) **Late markers:** GABAergic neurons (GAD1), Mature neurons (MAP2, NeuN, TUJ1), MSN (DARPP32, CTIP2), Late ventral forebrain (DLX5), Striasome (TAC1)	Striatal organoids used to examine the potential of HSF1 becoming a therapeutic target for treating Huntington’s Disease
Brady et al. (2022)	RT-qPCR, bulk RNA seq, IHC, Western blot	Five patient derived iPSCs, twelve healthy donor iPSCs	D8-11: Pur (2 µM) + FGF8 (200 ng/mL) D12-19: Pur (1 µM) + FGF8 (100 ng/mL) + RA (500 ng/mL) + BMP9 (10 ng/mL)	**Early markers:** Early MGE/POA (NKX2.1, CHAT, NCAD), Early ventral forebrain (DLX1/2, GLI1, SHH), Early CGE (PROX1) **Late markers:** Late LGE (FOXP1), D-2 MSN (DRD2), GABAergic neurons (GAD1), Striasome (PENK), MGE derived IN (NKX2.1, NOS-1, NPY, SST), Cholinergic IN (ISL1, CHAT, SLC18A3, SLC5A7), MSN (DARPP32)	Basal ganglia organoids displayed progenitors and neurons resembling LGE, MGE, and POA fates. Basal ganglia organoids reach up to 6 months *in vitro* and showed neurite extensions and dendritic spines with prolonged maturation
Sawada et al. (2023)	IHC, RT-qPCR, Western blot, MEA, scRNA-seq	Four patient derived iPSCs, four healthy donor iPSCs, MIN09i-33114.C control iPSC (WiCell)	D6-30: Pur (0.65 µM) + 100 ng DKK-1	**Early markers:** Early LGE (MEIS2), MGE (NKX2.1), CGE (NR2F1, NR2F2, SP8), Neural progenitors (SOX2, Ki67), Forebrain (FOXG1) **Late markers:** Late LGE (FOXP2, POU3F1), Developing MSN (CTIP2), Developing MSN2 (DARPP32, CALB1), Pre D1-MSN (TAC1, EBF1), Pre D2-MSN (SIX3, GRIK3), D1-MSN (IKZF1, PDYN, DRD1), D2-MSN (EGR3, PENK, ADORA2A, DRD2), Striatal INs (EPHB1, NKX2.1, EPHB3, CR), Cortical INs (NRP1, RELN, LHX6, HTR3A), Neocortex (EOMES, TBR1, SATB2), Astrocyte (GFAP, S100b)	Mature MSNs and INs were rarely detected in this protocol suggesting immaturity in ventral forebrain organoids. INs expressed marker genes for striosome and matrix. No unique cell types generated between D70 and D150. However, an upregulation of synaptic genes was observed in at D150. Additionally, increased synchronous activity and firing rate observed at D150 through MEA.
Kadoshima et al. (2013)	IHC	KhES-1 hESCs	D15-21: SAG (30 nM) Or D15-21: SAG (500 nM)	@SAG (30 nM) **Early markers:** Early LGE (GSX2), Forebrain (FOXG1), Neuroectoderm (PAX6) **Late marker:** GABAergic neuron (GAD2) @SAG (500 nM) **Early marker:** MGE (NKX2.1) **Late marker:** N/A	Observed a rolling NE morphology present primarily in the cortical PAX6+ regions of the organoid
Birey et al. (2017)	IHC, Calcium imaging, RT-qPCR, scRNA-seq, Whole cell patch clamp electrophysiology	Seven patient derived iPSCs, six control iPSCs from healthy donors, H20961 iPSCs (Gilad laboratory), H9 ESCs	D4-24: IWP-2 (4 µM) + EGF/FGF (10 ng/mL) D12-24: SAG (100 nM)	**Early markers:** Forebrain (FOXG1), MGE (NKX2.1), Early ventral forebrain (DLX1/2) **Late markers:** Cortical INs (SST, CR, CB, PV), GABAergic neuron (GAD1, GABA, SLC32A1, GPHN), Mature neurons (STMN2, DCX, MAP2, SYN1), Oligodendrocyte (OLIG2, SOX10), Choroid Plexus (TTR, SLC13A4), Late ventral forebrain (DLX5/6, NKX6.2), Migrating INs (ERBB4, NNAT, MALAT1, SOX11, NXPH1), Astrocyte (GFAP)	The addition of AlloP and RA significantly increased spontaneous calcium spiking but had no effect on the tissue specificity among subpallial organoids nor the GABAergic subtypes generated. Cortical-subpallial assembloid was used to investigate IN integration
Bagley et al. (2017)	IHC, RT-qPCR,	SC101A-1 Feeder-dependent iPSCs (Systems Biosciences), H9 ESCs (WiCell)	D5-12: SAG (100 nM) + IWP-2 (2.5 µM)	**Early markers:** Forebrain (FOXG1), Early ventral forebrain (DLX2), Early LGE (GSX2), MGE (NKX2.1, LHX6), Dorsal forebrain (PAX6, TBR1), Neural progenitors (Ki67), CGE (NR2F2, SP8) **Late markers:** GABAergic neuron (GAD1, SLC32A1), Mature neuron (HuC/D, NeuN, MAP2, DCX), INs (SST, NPY, CB, PV)	Used a cortical-subpallial assembloid and slice culture to catalogue IN migration behaviors. Noted that organoid migration undergoes a combination of radial and tangential migration mimicking the *in vivo* migration patterns of cortical INs
Xiang et al. (2017)	IHC, RT-qPCR, bulk RNA seq, scRNA-seq, Calcium Imaging, Whole cell patch clamp electrophysiology	HES3-NKX2-1-GFP hESCs, H1 hESCs (WiCell), 1090 iPSCs (donor derived)	D10-18: SHH (100 ng/mL) + Pur (1 µM)	**Early markers:** MGE (NKX2.1, NKX6.2, FOXA2, OLIG1/2), Forebrain (FOXG1), Neural progenitors (SOX2), Early ventral forebrain (DLX2, ASCL1), Neurogenesis (VIM, NES, HES1), Oligodendrocyte progenitors (S100A10, CD44) **Late markers:** GABAergic neurons (GAD1/2, GABA, SLC32A1), INs (SST, PV, NPY, CB, TAC1), Mature neurons (NeuN, STMN2, GAP43, TUJ1, DCX, SYN1, PSD95), Late ventral forebrain (DLX6), MGE derived IN (MAP2, ALCAM), Cortical derived IN (ZIC1, PTN, MEIS2), Migrating INs (CXCR4, NRP1) Oligodendrocyte (OLIG1/2), Astrocyte (GFAP, S100b)	Neurons from hMGEOs displayed calcium transients by D40-50. In large fields of view synchronous activity was documented. The electrophysiological properties of hMGEOs recorded action potentials in 8 out 14 cells. Fusion of hMGEOs and cortical organoids recapitulated cortical interneuron migration
Cao et al. (2022)	IHC, Calcium imaging, Whole cell patch clamp electrophysiology, organoid transplantation	IMR90-4 iPSCs (WiCell)	D10-25: SAG (1uM)	**Early markers:** MGE (NKX2.1), Forebrain (FOXG1), Neural progenitors (SOX2, Ki67, NESTIN, PKC-λ), Early dorsal forebrain (PAX6) **Late markers:** GABAergic neurons (GABA, GAD1), Mature neurons (DCX, NeuN, MAP2, TUJ1), INs (SST, CR, CB, nNOS), Astrocyte (GFAP)	Whole cell patch clamp revealed action potentials within hMGEOs starting at 6–8 weeks post differentiation and display electrophyisiological properties of mature neurons. Similarly, calcium transients in hMGEOs were significantly higher at D45 compared to earlier time points, again indicating neuronal maturation

## 2 Key morphogens for organoid ventralization

In general, forebrain organoid protocols utilize dual Smad inhibition for neural induction (Chambers et al., 2009). However, a guided differentiation approach requires additional signaling molecules for the subsequent controlled tissue patterning of organoids. Here we review different ventralizing molecules that activate and influence telencephalic patterning (Figure 1C).

### 2.1 SHH

In organoid cultures, the Shh pathway is commonly activated using recombinant Shh protein and/or smoothened agonist-targeting small molecules such as Purmorphamine (Pur) or Smoothened Agonist (SAG). Doing so mimics the endogenous subpallial cues that induce ventral telencephalic patterning. Modulating the concentration and timing of Shh pathway activation leads to varying degrees of ventralization with the higher concentrations and longer durations corresponding with the production of more ventral GE progenitors. For example, many striatal organoid protocols will use a lower concentration of Pur/SAG or recombinant SHH to induce a more dorsal GE (dGE) fate. In doing so, they enrich LGE-like progenitors which are the biggest contributors for the development of the striatum. On the other hand, increasing the degree of SHH pathway activation leads to more ventral GE fates, specifically the MGE.

Interestingly, the type of agonism chosen seems to play a role in how effective ventralization will be. For example, recombinant Shh proteins tend to be less effective for ventralization and this might be attributed to its shorter half-life and stability compared to its small molecule counterparts, Pur and SAG (Krajka et al., 2021). In 2D cell cultures, Pur has been shown to efficiently induce ventral fates in cells (Amimoto et al., 2021; Krajka et al., 2021). However, Pur has a half-maximal effective concentration in the micro molar range for SMO activation (Sinha and Chen, 2006). On the other hand, SAG has a half-maximal effective concentration in the low nanomolar range and has the property of inducing high degree of ventralization with less material (Chen et al., 2002; Wang et al., 2010; Wu et al., 2018; Shen et al., 2020). A final consideration is that a water soluble reagent like SAG might reduce the potential for introducing a stressor, as is the case with DMSO-soluble Pur.

### 2.2 SAG

Kadoshima et al., 2013 was among the first to attempt targeted ventralization of a neural organoid using 30 nM of SAG to generate an LGE lineage of progenitors expressing markers such as FOXG1, GSX2, and PAX6. When attempting a higher concentration of SAG, 500nM, the progenitors of the neuroepithelium domain largely expressed the canonical MGE marker NKX2.1, indicating a greater degree of ventralization in the organoid tissue corresponding to the increased SAG dosage. To expand on this finding, Birey et al., 2017 developed a subpallial protocol using 100 nM of SAG (Days 12–24 *in vitro*) that showed high expression of NKX2.1 and FOXG1 present within rosette progenitors of 25-day old organoids. Gene expression level of subpallial organoids at day 60 possibly hints at a more diverse pool of ventral neural progenitors with DLX1/2 expression. Similarly, Bagley et al., 2017 also used 100 nM SAG [Days 5–12], which again resulted in high FOXG1 expression confirmed through qPCR analysis. Interestingly, they reported a mix of DLX2, GSX2, NKX2.1, and LHX6 expression within their Day 30–40 ventral organoids. These initial findings indicate a more diverse mixture of GE progenitors. Indeed, further analysis suggested the presence of LGE/CGE derived cell types amongst the differentiated GABAergic cell types. While Birey et al., 2017 and Bagley et al., 2017, used identical concentrations of SAG, the duration of exposure and the specific use of additional small molecules/growth factors likely played a role in the final neuronal specification of their organoids (Table 1). Finally, Cao et al., 2022 opted for a significantly higher concentration SAG at 1uM, aiming to specify their organoids towards an MGE lineage. They did in fact attain a strong expression of NKX2.1 in 93% of cells by Day 30. Furthermore, progenitor markers SOX2 and Nestin were seen alongside FOXG1, specifically indicating the presence of a ventral forebrain neural progenitor pool.

### 2.3 Pur + rShh

Xiang et al., 2017 took a different approach, opting to use a combination of 100 ng/mL of recombinant SHH with 1uM Pur to supply a ventral rich environment. With this approach, NKX2.1 expression was first reported using the HES3-NKX2-1-GFP reporter line as early as 3 days after starting SHH exposure (Day 13). By Day 18, NKX2.1 expression thoroughly enveloped the entirety of the organoid, indicating a robust generation of MGE-like progenitors. By Day 21, ∼82% of cells expressed NKX2.1 and about 93% of cells expressed FOXG1. On the other hand, with the sole addition of 200 ng/mL of recombinant SHH, Liu et al., 2022 was able to generate striatal organoids, which generated GSX2 expressing progenitors of VZ rosette structures. This was based on the Ma et al., 2012 striatal protocol, which reported a comparable effect of 0.65uM Pur when compared to 200 ng/mL of recombinant SHH for inducing LGE-like progenitors.

### 2.4 Pur

Other protocols have followed suit and adopted similar methodologies for striatal development in 3D. Adil et al., 2018 initially formed 3D hESC aggregates in a hydrogel matrix exposed to 0.65uM of Pur and successfully induced LGE progenitors expressing GSX2 and OTX2 at Day 26. Alternatively, Amimoto et al., 2021, recapitulated similar results using a slightly lower 0.5uM Pur, but still cataloged wide expression of the forebrain marker FOXG1 and LGE markers GSX2 and DLX2 in their organoids as early as Day 20. Chen et al., 2022 striatal organoids were induced using 0.65uM Pur and at Day 20 displayed a high yield of PAX6 and SOX1 double positive cells with ∼85% of cells also positive for OTX2 and FOXG1. Minimal NKX2.1 expression was detected within the 0.65uM Pur conditions, but by Day 45, immunostaining data revealed a high expression of LGE markers GSX2, DLX2, and MEIS1/2 within rosettes. Most recently, Sawada et al., 2023 used 0.65uM Pur concentration and developed ventral forebrain organoids that displayed progenitors that had SOX2 co-expressed with MEIS2, NR2F2, and NKX2.1 showing progenitors from all 3 GE lineages by Day 37. As previously alluded to, the modulation of SHH pathway activation is an important factor for the subpallial patterning of forebrain organoids, but there are other variables at play that can regulate the ultimate differentiation of the organoid.

### 2.5 WNT

Both the Dorsal-Ventral (DV) and Anterior-Posterior (AP) axis patterning rely on WNT gradients produced by the surrounding tissue (Maretto et al., 2003; Harrison-Uy and Pleasure, 2012). Many forebrain organoid protocols include some form of WNT inhibition (WNTi) during neural induction to enrich for anterior progenitors. During neural differentiation, WNTi might once again be applied in combination with Shh agonism to push tissue towards a more ventral fate. The Wnt signaling pathway has been shown to play a strong role in regulating ventral patterning (Tole et al., 2000; Kuschel et al., 2003). The loss of beta-catenin, specifically in early neurodevelopment, displayed a tendency for increased expression of ventral markers in cortical progenitor cells (Backman et al., 2005). Concomitantly, when the Wnt signal is disrupted in mice, ventral markers will extend dorsally (Yu et al., 2008). The dynamic role of Wnt, is not restricted to patterning-like effects on the subpallium but can also influence ventral proliferation. For example, Wnt signaling in the ventral forebrain has been shown to regulate the expansion of progenitor cells of the MGE (Gulacsi and Anderson, 2008). Here we have highlighted the necessary considerations to account for when modulating the Wnt pathways *in vitro*.

Common small molecules for WNTi include XAV939 and IWP-2, but recombinant Dickkopf-1 (DKK-1) serves as an alternative. XAV939 works intracellularly to stabilize Axin by inhibiting tankyrase enzymes and promoting the degradation of beta-catenin and blocking the WNT signaling cascade. IWP-2, on the other hand, inhibits the enzyme porcupine, which in turn halts the extracellular secretion of the WNT ligand. DKK-1 inhibits the WNT pathway by binding to the co-receptor LRP5/6 and thus blocking the initial interaction with WNT. In the subpallial protocols, multiple methods of WNTi are used in combination with SHH agonism for varied results (Table 1).

### 2.6 FGF

While the Shh pathway is primarily targeted in most subpallial protocols, Shh indirectly causes ventralization. This is because the role of Shh is to inhibit the dorsalizing factor Gli3. Gli3 normally inhibits FGF expression, therefore in the presence of Shh, FGFs are responsible for the ventral patterning of tissue in the telencephalon (Gutin et al., 2006; Storm et al., 2006; Hébert and Fishell, 2008). The complexity of gene regulation strongly implicates FOXG1 and FGFs in the ventralization of the telencephalon; however, both also seem to interact with PAX6 for the delineation of the dorsal telencephalon (Hébert and Fishell, 2008). This effect mirrors that of the role of Wnt on the proliferation of MGE progenitors (Gulacsi and Anderson, 2008). These morphogenic effects are further emphasized by the work done in Shh and Gli3 KO mice. First, when Shh is knocked out in mice, the ventral telencephalon fails to develop normally with extension of PAX6 and EMX1/2 cortical populations into the subpallium. The Gli3 KO mice show the opposite effect with stunted cortical development and the dorsally expanded subpallium. However, Shh and Gli3 double KO mice display a rescued D-V phenotype where the cortex and subpallium more closely resemble the typical axes of polarity of a control mouse and leads to the re-establishment of NKX2.1 expression in the MGE (Rallu et al., 2002). Further still, FGFs, and particularly FGF8, play key roles in establishing the anterior forebrain early in development with a large FGF source coming from the Anterior Neural Ridge (ANR) (Shimamura and Rubenstein, 1997; Houart et al., 1998). Removal of the ANR results in dramatic loss of the telencephalon with the diencephalon remaining as the sole forebrain structure. The implantation of an FGF8 expressing bead in place of the ANR can induce FOXG1 expression and partially rescue A-P patterning of the early neural plate (Shimamura and Rubenstein, 1997). Given the dynamic role of the FGF family, and its stage-dependent effect on the neuroectoderm, timing of exposure and combination with other small molecules should be closely considered before implementation into organoid patterning.

### 2.7 Retinoic acid (RA)

Normally considered as a caudalizing morphogen, exogenous RA promotes spinal cord specification in hESC derived neuroectoderm (Gavalas and Krumlauf, 2000; Wichterle et al., 2002; Chambers et al., 2009). However, the role of RA in telencephalic patterning remains opaque with most studies relegating the role of RA to LGE maturation/maintenance and cortical expansion (Molotkova et al., 2007; Chatzi et al., 2013). In turn, RA activity within the LGE is key to establishing GABAergic differentiation within the basal ganglia/striatum (Chatzi et al., 2011). Therefore, certain organoid protocols looking to establish a basal ganglia/striatal phenotype have manipulated RA concentrations within their cultures. For example, in addition to Shh, FGF, and BMP9, Brady et al., 2022 utilized 500 ng/mL of RA to derive basal ganglia organoids composed of progenitors and neurons resembling LGE, MGE, and POA fates. Uniquely, at Day 23 and Day 45, over 55% of cells in the basal ganglia organoids expressed NKX2.1 with about 20% of the progenitors co-expressed NKX2.1 and CHAT. Additionally, 20% of cells expressed the pan-GE markers DLX1/2. Interestingly, the transcriptomic profile of basal ganglia organoids was similar to that of the fetal ventral telencephalon. In parallel, Miura et al., 2020 identified SR11237 as a small molecule capable of modulating the retinoic acid receptor pathway (RXRG) and used it in combination with Activin A and IWP-2 to guide the forebrain organoid towards striatal fates. Activin A has previously been shown to assist in MSN generation (Arber et al., 2015). The use of SR11237 was a unique approach that circumvented the need to manipulate the SHH pathway and therefore limited the carryover from MGE-like cells to 0.002%. Miura’s striatal organoids displayed GSX2 and CTIP2 by Day 15 and high levels of FOXG1, DLX2, GSX2, and MEIS2 by Day 22. A recent study by Barmpa et al. (2023) followed up on the use of SR11237 for striatal organoid protocols by comparing the Miura protocol to modified subpallial protocols adding RA/SAG or SR11237/SAG. While all protocols seemingly establish striatal identity within their organoids, those exposed to SAG displayed prolonged maturation due to a larger retention of progenitors up to Day 50. In contrast, the Miura protocol, overall displayed higher levels of GSX2, FOXP1, FOXP2, and CTIP2, DARPP32, and DRD1.

## 3 Striatal GABAergic neurons generated from subpallial organoids

### 3.1 Medium Spiny Neurons (MSNs)

The GABAergic projection neurons of the striatum, MSNs, are amongst the most experimentally exploited group of cells for subpallial organoids. Dysfunction in MSNs has been implicated in neurodegenerative disease such as Huntington’s and Parkinson’s disease. MSNs make up 90% of the cells in the striatum with the other 10% coming from GE derived INs (Kawaguchi, 1997). Specifically, the ISL1 expressing progenitors of the ventral LGE are primarily responsible for most of the MSN generation (Stenman et al., 2003). Mature MSNs can be further subdivided by their expression of the D1 dopamine receptor (D1DR) or D2 dopamine receptor (D2DR) (Gerfen et al., 1990). In the direct pathway, GABAergic projections from striatal D1DR MSNs project directly onto Globus Pallidus Interna (GPi) and Substantia Nigra Pars Reticulata (SNr) effectively leading to the disinhibition of thalamocortical motor circuits. On the other hand, the indirect pathway requires D2DR MSNs synapsing onto the Globus Pallidus Externa (GPe) in turn decreases inhibition of the SubThalamic Nucleus (STN) which has cascading effects leading to inhibition of thalamocortical circuits. A second level of compartmental organization persists within the striatum encompassed by the separation of MSNs into striosomal patch and matrix compartments (Gerfen, 1992). Both the striosome patch and matrix differ in their neurochemical composition as well as their connectivity patterns (Crittenden and Graybiel, 2011; Fujiyama et al., 2011). Despite the observable diversities, mature MSNs can be keenly identified by the co-expression of CTIP2 and DARPP32 (Arlotta et al., 2008).

### 3.2 Pur patterned striatal-like organoids

As previously described, Pur as a SHH pathway agonist is quite potent for inducing LGE progenitors which later go on to produce MSNs. Likewise, striatal organoid protocols tend to use the 0.65uM Pur concentration to induce striatal fates in 3D. While different protocols also aim to inhibit the WNT pathway to improve MSN yield, a relatively lower concentration of SHH agonism seems to be consistent. For example, when 3D aggregates were cultured in with 0.65uM Pur and 100 ng/mL of DKK-1 for 26 days and allowed to differentiate in 2D for 60 days, Adil et al., 2018 found that 77% of cells in culture contained the mature neuronal marker MAP2. Of the MAP2 containing cells, 55% were DARPP32 and CTIP2 double positive. Taking a similar approach in 2D, Amimoto et al., 2021 used a slightly lower 0.5uM Pur concentration and reported ∼60% DARRP32/GABA expression in their cells with many co-expressing CTIP2 (quantification not available). A similar effect was seen in their Day 96 organoids with DARPP32 and CTIP2 co-expression confirmed through IHC. Chen et al., 2022 relied solely on 0.65uM of Pur for their striatal patterning and reported about 18.27% of cells in their Day 100 organoids co-expressing CTIP2/DARPP32/NeuN, indicating the presence of mature MSNs. The ventral forebrain organoids from Sawada et al., 2023 used 0.65uM Pur with 100 ng/mL of DKK-1 for a total period of 25 days. When cultured long term for upwards of 70 and 150 days, an average of 67.4% of cells were inhibitory cell types with some pre-MSNs (MEIS2/POUF3F1) detected amongst this population. Still, they were able to distinguish both pre-D1 (TAC1/EBF1) and pre-D2 (SIX3/GRIK3) MSNs present within their ventral organoids.

As the name suggests, the basal ganglia organoids described in Brady et al. (2022) contained a large component of striatal tissue with significant upregulation of striatal/MSN markers DARPP32 and DRD1 when compared to control cortical organoids. However, the basal ganglia organoid is distinct as the protocol requires a high concentration pulse of Pur (2uM) and FGF8 (200 ng/mL) for 3 days initially which then transitions to 1uM Pur and 100 ng/mL FGF8 for the remaining 7 days in combination with 500 ng/mL RA and 10 ng/mL of BMP9. This morphogen cocktail primarily models the early GE and POA progenitors in culture; and once mature, the organoids host an interesting diversity of MGE-derived interneurons and cholinergic interneurons as well.

### 3.3 Recombinant SHH patterned striatal-like organoids

With a comparable effect to that of 0.65uM Pur, Liu et al., 2022 used recombinant SHH at 200 ng/mL to generate mature MSNs. At Day 60, DARPP32, CTIP2, and NeuN were observed outside of rosette regions, although co-expression was not confirmed. Still, single cell transcriptomics annotated a group of MSNs at Day 30 that increased in Day 60 organoids.

### 3.4 RA striatal organoids

When targeting the RXRG receptor using SR11237, Miura’s (2020) striatal organoids can mature up to ∼ Day 80 and confirm co-expression of DARPP32 and CTIP2 through IHC. Seemingly, ∼50% of cells expressed CTIP2 with ∼10% expressing DARPP32. A deeper comparison of RA/SAG and SR11237 protocols revealed similar abilities to induce striatal fates with accelerated maturation observed in protocols targeting the RXRG receptor as assessed by higher expression of DARPP32, DRD1 and GAD65 in Day 50 organoids (Barmpa et al., 2023).

## 4 Forebrain interneuron subtypes generated from subpallial organoid protocols

Based on the aforementioned patterning agents and timing, different groups have generated subpallial organoids with specific goals to enrich for specific cell types, i. e., striatal organoids generating MSNs, as mentioned above. Still, the excitatory/inhibitory (E/I) balance of cortical networks is primarily made up of glutamatergic neurons and GABAergic interneurons. Given that a large majority of cortical interneurons are MGE/CGE derived, the reported IN yields from subpallial organoid protocols is wide ranging. In the following section, we discuss the most prominent IN subtypes and their reported appearance in organoids cultures. Overall, to date, the most commonly reported IN subtypes are SST+ and CR+ (CALB2) neurons. While all IN subtypes listed below originate from GE progenitor pools, it is unclear as to why only a subset of INs are generated within organoid cultures. The observed variability brings up the question of whether additional factors might be necessary for wider IN representation, such as activity dependence, extracellular matrix, migration cues, or even the presence of a dorsal pallium for INs to integrate into (Antonopoulos et al., 1992; Götz and Bolz, 1994; Mione et al., 1994; Obst et al., 1998; Bartolini et al., 2013).

### 4.1 Somatostatin (SST) interneurons

While the MGE is characterized by VZ progenitors expressing NKX2.1, the postmitotic neurons of the MGE go on to populate a large majority of the INs in the cortex. Developmentally, the MGE, particularly the dorsal MGE (dMGE), preferentially generates SST + INs earlier than PV + INs (Flames et al., 2007; Wonders et al., 2008). *In vitro*, many subpallial organoids have successfully exhibited the ability to recapitulate this temporal regulation (Bagley et al., 2017; Birey et al., 2017; Xiang et al., 2017). Interestingly, Xiang et al., 2017 showed that increasing the concentration of SHH in organoid cultures seemingly increased the production of SST + INs. It is important to note that, *in vivo*, although the dMGE is seemingly further away from a SHH source, the dMGE contains an increased expression of SHH signaling effectors which could make it far more sensitive to SHH signaling (Wonders et al., 2008; Yu et al., 2009). Similarly, in slice culture, exposing the ventral MGE (vMGE) to exogenous SHH suppresses its preference for making PV + INs in favor of SST + fates (Xu et al., 2010). This might provide a potential explanation for the observations made by Xiang et al., 2017.

### 4.2 Parvalbumin (PV) interneurons

The fast-spiking PV interneurons are characteristically born from the MGE. This subtype of INs make up a majority of the INs in the human cortex (Gonchar and Burkhalter, 1999; Markram et al., 2004; Bodor et al., 2005). However, given their developmentally late onset of generation in the fetal telencephalon, organoid counterparts have been elusive and require longer culturing periods before they can be reliably observed (Bagley et al., 2017; Birey et al., 2017; Xiang et al., 2017; Sloan et al., 2018). Despite the strong prevalence of PV INs in the human cortex, subpallial organoids have struggled to reliably increase the yield of PV + INs through morphogen manipulation.

### 4.3 Vasoactive intestinal polypeptide (VIP) interneurons

The genetic signatures of the ventral-caudal CGE bear much resemblance to the LGE. Progenitors in both of these regions share expression of key markers such as NR2F2, GSX2, MEIS2, and PAX6 (Yun et al., 2001; Frazer et al., 2017). However, CGE derived INs will go on to migrate into the cortex while LGE derived INs are fated for the OB. Further still, the CGE uniquely produces the disinhibitory VIP INs that synapse onto other cortical INs (Rudy et al., 2011; Tremblay et al., 2016; Huang and Paul, 2019). Developmentally, CGE derived INs are produced at a later time point compared to MGE derived INs (Miyoshi et al., 2007; 2010). As previously noted, even the later born PV INs can take well up to 100+ days to robustly, if at all, appear in subpallial organoids. Given the late onset of VIP neurons, it is no surprise that VIP + INs have been rarely reported in telencephalic organoids (Sawada et al., 2023). Nonetheless, various protocols have reported CGE-like progenitors in their cultures expressing NR2F2, SP8, PROX1, and PRKCA (Bagley et al., 2017; Birey et al., 2017; Gabriel et al., 2021; Brady et al., 2022; Andrews et al., 2023; Pavon et al., 2023; Sawada et al., 2023). Perhaps given the right temporal culturing conditions and the correct environmental cues, the differentiation of the CGE-like progenitors into mature INs might be achievable.

### 4.4 ID2/Lamp5 interneurons

Formerly characterized as Reelin (RELN)+ INs, the ID2 INs are CGE derived (Mayer et al., 2018; Fishell and Kepecs, 2020). Both ID2+ and VIP + INs, together, make up ∼30% of all cortical interneurons and are produced by the CGE at similar ratios (Miyoshi et al., 2010). Despite the significant percentage of IN populations that the CGE contributes, subpallial organoids rarely exhibit signs of mature CGE derived INs.

### 4.5 Calretinin (CR) interneurons

The calcium binding protein calretinin (CR; CALB2) serves as the primary marker for a small portion of cortical INs thought to be derived from the CGE. Still, the CR marker has also been shown to commonly co-express in VIP+ and, to a lesser extent, SST + INs (Cauli et al., 2014; Lim et al., 2018a). In addition to the cortically fated CR INs that are CGE derived, the dLGE has also been shown to generate SP8+ neuroblasts that migrate through the RMS and partially contribute to the CR + INs of the granule cell layer of the OB (Waclaw et al., 2006). The expression of CR + INs has been commonly reported in many subpallial protocols [Table 1]; however, their subtype diversity and dynamic migration keeps them an elusive target for characterization.

## 5 The default patterning of GABAergic interneurons in cortical organoid protocols

One of the most fascinating aspects of cortical forebrain protocols is the production of GABAergic cells in both default non-directed and guided differentiated organoids (Quadrato et al., 2017; Velasco et al., 2019; Tanaka et al., 2020; Magni et al., 2022). It has been speculated that the primate cortical progenitors can produce these GABAergic INs, which is in stark contrast to the rodent data (Anderson et al., 1997; Letinic et al., 2002; Cunningham et al., 2013; Radonjić et al., 2014; Al-Jaberi et al., 2015; Clowry, 2015). Alternatively, the analysis of default patterning protocols in 2D, which forms the basis for unguided organoid protocols, show that many of the cortical development models report inhibitory signals within their cultures (Weick et al., 2013; Larsen et al., 2016).

Floruta et al., 2017., showed that upon closer inspection, the seemingly dorsal PAX6+ progenitors of many cortical protocols share characteristics with the progenitors of the CGE/LGE, such as DLX1/2, ARX, SP8, and ASCL1. Immunostaining in default patterned protocols confirmed DLX and NR2F2 expression, as well as the mature interneuron marker CR.

Further examination of default patterned neuronal cultures showed gene expression overlap with the *in vivo* cortex and striatum, again hinting at a mixed population of cell types. Additionally, Floruta et al., 2017 compared guided differentiation protocols and noted a successful increase of cortical gene expression when incorporating WNT antagonism or the addition of EGF/FGF2 (Watanabe et al., 2005; Mariani et al., 2012; Paşca et al., 2015). The adoption of WNT inhibitor DKK-1 did seem to selectively improve cortical specification. Conversely, the addition of EGF/FGF2 improved cortical fates, but also increased cerebellar gene expression as well. This finding was complemented by the large multiplexed morphogen screening in organoids performed by the Pasca lab, which again showed the effects of EGF/FGF2 in stimulating cerebellar gene expression (Amin et al., 2023). Further still, FGF2 has displayed a posteriorizing effect during the neuralization of hESCs by promoting a hindbrain fate (Lupo et al., 2013). While FGF2 is commonly added to culture media for inducing its mitogenic properties, it is important to note that FGF2 has a less specific receptor binding affinity (Ornitz et al., 1996; Zhang et al., 2006; Kwiatkowski et al., 2008). This means that FGF2 can potentially bind with a wide variety of FGF receptors including those which might activate morphogenic pathways.

It is worth noting that a study by Pappas and Parnavelas 1998 displayed increased proliferation of GABAergic CR neurons from cortical progenitors in response to FGF2. Yung et al., 2002 showed that clonally derived and FGF2 expanded, E16.5 cortical cultures, are capable of giving rise to GABAergic neurons. However, early embryonic FGF2 expanded cortical cultures (E12.5), were almost entirely glutamatergic and only ventralized in the presence of SHH. Gabay et al., 2003 elegantly displayed the ability of FGF2 to induce the expression of the ventral Olig2 marker in dorsal progenitors. Fascinatingly, FGF2 seemed to increase endogenous SHH measured in dorsal cultures with an increase of SHH mRNA. Altogether, it is worth recognizing that the diversity of cell types generated in cortical organoids can be influenced by the choice of morphogens, growth factors, and timings. Using them in certain combinations might lead to the creation of an unintended environment primed for a ventral progenitor domain, which can be exacerbated with prolonged morphogen/growth factor exposure.

## 6 Challenges with current methods in generating mature GABAergic neurons

The qualities of interneuron specification remain relatively unknown. One theory suggests that INs differentiate specifically as a response to the environmental cues they receive during migration or at their settling point (Stumm et al., 2003; Tiveron et al., 2006; Bortone and Polleux, 2009; Bartolini et al., 2013). Indeed, many INs do not express their characteristic genetic signatures until they have reached their endpoint in the cortex (Marín and Rubenstein, 2001; Miyoshi et al., 2010; Kepecs and Fishell, 2014; Wamsley and Fishell, 2017; Lim et al., 2018a). Other lines of evidence suggest that cell specification occurs as early as the progenitor stage and can contain an element of temporal influence (Lim et al., 2018b; Mayer et al., 2018).

This provides a challenge in subpallial organoids that are homogeneously exposed to SHH agonists. Doing so limits the generation of fully cortical environments for these INs to successfully integrate into and to form functional synaptic connections in a region-specific manner. As seen in the subpallial protocols examined in this review, targeted IN generation is not a straightforward approach. If one wishes to improve the developmental modeling of forebrain organoids, it will be critical to enhance GE progenitor pools that can yield IN subtypes that better capture the IN diversity observed *in vivo*.

## 7 Future direction

**FIGURE 3:**
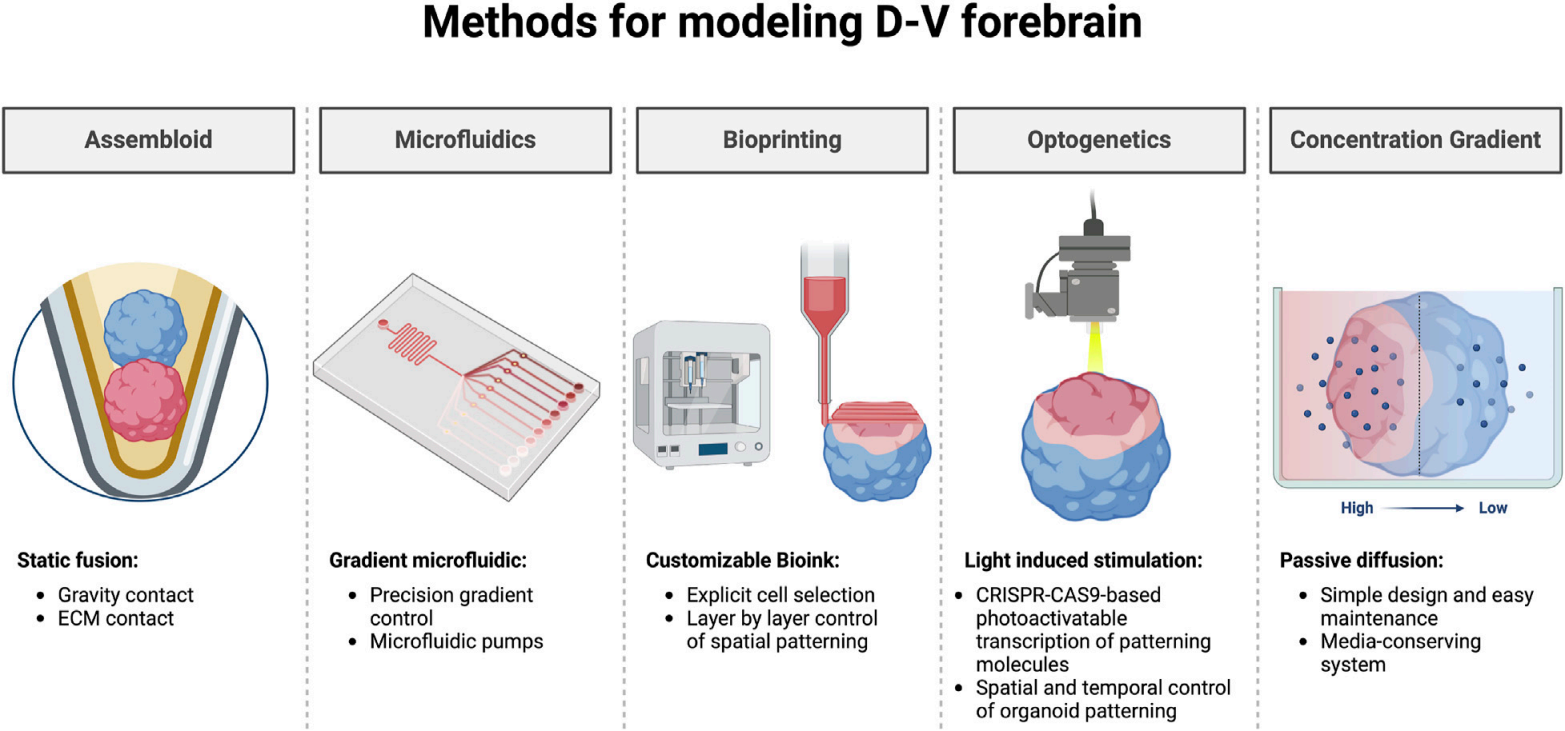
Bioengineered methods for dorsoventral patterning of the human forebrain. Exciting prospects for the spatiotemporal patterning of forebrain organoids. Regionally specified neural organoids are commonly fused to form assembloids using a static approach relying on gravity or ECM to establish prolonged tissue contact. Microfluidics offer highly tunable environments capable of establishing morphogen gradients *in vitro*. Using specified bioink, 3D bioprinting could provide a method for generating organoids with controlled spatial patterning. Light induced stimulation not only provides an avenue for manipulating neural activity, but also for the photoactivatable transcription of key patterning molecules for control of spatiotemporal patterning. Establishing a morphogen concentration gradient by leveraging passive diffusion offers an easy-to-use platform capable of inducing tissue patterning within neural organoids. Created by using Biorender.com

Currently, the assembloid model aims to mitigate the lack of cortical environment by fusing cortical organoids with subpallial organoids. This assembloid method has efficiently stimulated IN migration and integration from subpallial organoids into cortical organoids, thus allowing for improved characterization of subpallial organoid derived INs (Bagley et al., 2017; Birey et al., 2017; Xiang et al., 2017). Indeed, striatal-midbrain and cortico-striatal organoid fusion have displayed increased neuronal maturity (Xiang et al., 2017; 2019; Barmpa et al., 2023). However, the assembloid approach requires two separate protocols for each organoid type and does not fully recapitulate the early neural patterning by failing to reproduce the cohesive transition established by structural intermediates. Alternative approaches focus on the establishment of a morphogen gradient to reproduce the distinctiveness of early progenitor pools across the D-V and A-P axis. For example, Cederquist et al., 2019 integrated a SHH expressing aggregate to their forebrain organoids which effectively patterned MGE (NKX2.1), LGE (GSX2), and cortical (PAX6) progenitors within a single organoid (Cederquist et al., 2019). Currently, the integration of bioengineering and microfluidics has already shown success for tissue patterning using controlled morphogen diffusion (Cosson and Lutolf, 2014; Demers et al., 2016; Kim et al., 2018; Rifes et al., 2020). Recently, the author’s lab has shown the ability to establish D-V patterning of the GEs in forebrain organoids using the passive diffusion of Pur in a Morphogen-gradient Induced Brain Organoid system (MIBOs) (Pavon et al., 2023). MIBOs generate dorsal GE-derived CR + INs, MSNs, and medial GE-derived cell types within a single organoid and can be further finetuned with variations in morphogens, concentrations, and durations. Further still, the ingenious adoption of optogenetic stimulation for spatially controlled Shh gene expression within a neural organoid has also shown promise for subpallial tissue patterning (Legnini et al., 2023). With the advent of 3D bioprinting, functional and patterned neural tissue can already be developed, adding a potential tool for the further improvement of organoid technologies (Yan et al., 2024). The exciting prospects of incorporating further engineering techniques for organoid development suggest that our ability to model the fetal forebrain is exceedingly promising (Figure 3).

Beyond tissue patterning, bioengineering has the potential to enhance both the structural integrity, and functional properties of organoids. Some groups have incorporated endothelial cells during organoid formation for the vascularization of neural organoids, adding a new dimension for studying the circulatory system and mitigating organoid necrotic cores (Pham et al., 2018; Shi et al., 2020). On the other hand, both optogenetics and electrical stimulation can be leveraged to manipulate the neural activity of organoids leading to improved circuit formation, maturation, and functionality (Quadrato et al., 2017; Meng et al., 2023). Moreover, the precision control and streamlined automation of 3D bioprinting offer exciting prospects for the D-V patterning of forebrain tissue (Gao et al., 2021). While current fusion approaches have shown great promise for improved maturation within organoids, alternative approaches such as morphogen gradient induction or bioengineering methodology offer the potential of creating a more comprehensive model of the developing telencephalon (Figure 3). Further efforts to induce opposing morphogenic gradients simultaneously to deliver complex gradient schemes to recapitulate early neural tube patterning will undoubtedly propel the field forward.

The advent of subpallial organoids cannot be understated as they provide crucial insight into the development of GABAergic cell types. Specifically, the assembloid models have been particularly useful for examining the mechanisms of interneuron migration in human cellular context (Xiang et al., 2019; Birey and Pasca, 2022; Roth et al., 2023). Neurodevelopmental, as well as neurodegenerative, disease pathology, has shown to be specifically impact the IN populations of the cortex (Brunet et al., 2020; Crabé et al., 2020; Xu et al., 2020; Cai et al., 2021). Such is the case in schizophrenia where the excitatory/inhibitory (E/I) balance of the prefrontal cortex and the hippocampus are particularly disrupted in patients (Liu et al., 2021; Uliana et al., 2024). Likewise, in Huntington’s disease, the misfolding and aggregation of the mutant Huntingtin Protein (mHTT) leads to the progressive loss of MSNs that results in the characteristic motor symptoms as well as cognitive deficits seen in patients (Han et al., 2010; Ehrlich, 2012; Bunner and Rebec, 2016). Not only do subpallial organoids provide the opportunity to examine the development of GABAergic neurons in disease states, but they also give us a platform for improved pharmaceutical assays. As previously shown, 3D tissue culture exhibit ECM interactions which better models tissue abnormality and can be advantageous for drug toxicity screenings specific to humans (Langhans, 2018; Zhou et al., 2023). To date, many groups have used neural organoids to study the compound effects on developing neural tissue (Lee et al., 2017; Arzua et al., 2020; Dang et al., 2021). One group has even used midbrain organoids for a high throughput toxicity screening of 84 compounds further punctuating the effectiveness of neural organoids for pharmacological testing (Renner et al., 2021). The continued refinement and adaptation of subpallial organoid models will be instrumental in advancing our understanding of human fetal subpallium development, with far-reaching potential for future discoveries. As such, the incorporation of a 3D neural model as a tool for drug discovery holds great promise for the future personalized medicine in patient populations.
